# Topical application of L-arginine blocks advanced glycation by ascorbic acid in the lens of hSVCT2 transgenic mice

**Published:** 2011-08-18

**Authors:** Xingjun Fan, Liu Xiaoqin, Breshey Potts, Christopher M. Strauch, Ina Nemet, Vincent M. Monnier

**Affiliations:** 1 Department of Pathology, Case Western Reserve University, Cleveland, OH; 2 Department of Biochemistry, Case Western Reserve University, Cleveland, OH; 3 John F. Kennedy High School, Cleveland, OH

## Abstract

**Purpose:**

Previous experiments from our laboratory showed that the oral intake of selected guanidino compounds could block the formation of crystallin-bound advanced ascorbylation products. Here we tested whether these were also active when applied as eye drops.

**Methods:**

Two month old hSVCT2 transgenic mice (n=10) were treated twice daily with one drop of 0.1% L-arginine, γ-guanidinobutyric acid (GBA), penicillamine (PA) or *N*-acetylcysteine (NAC) in one eye and vehicle only in the other eye. After seven months, lens crystallins were isolated, dialyzed, and proteolytically digested to determine the protein-bound fluorescence at 335/385 and 370/440 nm excitation/emission and the advanced glycation/ascorbylation endproducts carboxymethyl-lysine (CML), carboxyethyl-lysine (CEL), glucosepane, glyoxal, and methylglyoxal hydroimidazolones G-H1 and MG-H1. The topical uptake of L-arginine and NAC was also evaluated in vitro and in vivo in rabbit lens.

**Results:**

In hSVCT2 mice, L-arginine decreased 335/385 and 370/440 nm fluorescence by 40% (p<0.001), CML, CEL, and glucosepane crystallin crosslinks by 35% (p<0.05), 30% (p<0.05), and 37% (p<0.05), respectively, without affecting MG-H1 and G-H1. NAC decreased 335/385 nm fluorescence by 50% (p<0.001) but, like PA and GBA, had no effect on other modifications. L-Arginine uptake into rabbit eyes treated topically reached identical lenticular plateau levels (~400 nmol/g wet weight) at 0.5% and 2.0% but levels remained three times higher at 5 h at 2% versus 0.5% concentration, respectively. In vitro studies showed a 100 fold higher L-arginine level than NAC levels, implicating high affinity uptake of the former.

**Conclusions:**

L-Arginine when applied both orally and topically is a potent and broad suppressor of advanced ascorbylation in the lens. Its uptake in rabbit lens upon topical application suggests transcorneal uptake into the human lens should be feasible for testing its potential anticataract properties in clinical trials.

## Introduction

Aging human lens crystallins accumulate several modifications which include the formation of protein disulfides, oxidation of methionine residues, protein fragmentation and cross-linking by disulfide and non-disulfide bonds, deamination, deamidation and accumulation of colored and colorless, fluorescent or non-fluorescent products [1]. The significance of these modifications is that they can destabilize lens crystallins, impair their chaperone function, unfold the protein, and increase their susceptibility toward oxidation and aggregation, eventually leading to the formation of high molecular weight products that are opaque, i.e., cataractous.

**Figure 1:**
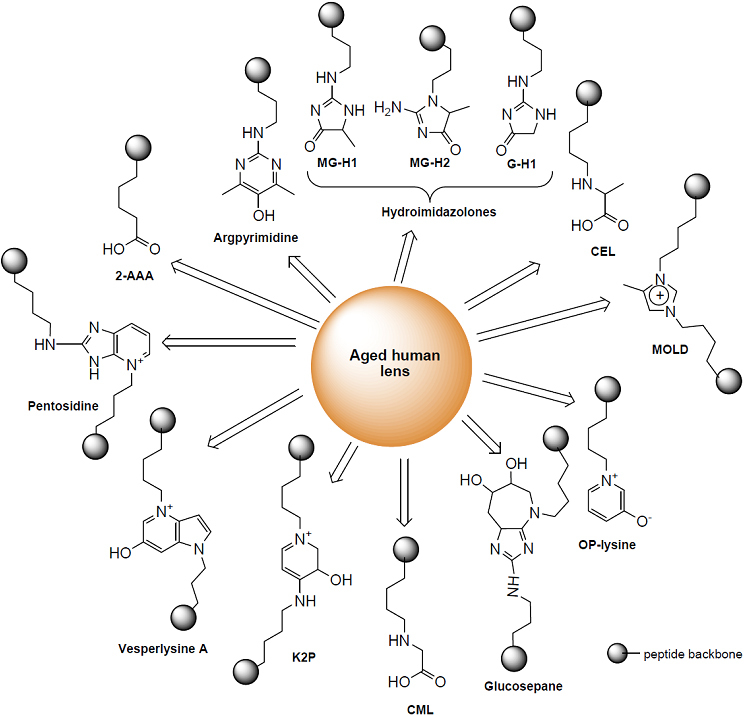
Structure of AGEs identified in the aging human lens.

Among these modifications, our laboratory has obtained strong evidence for the hypothesis that ascorbic acid oxidation products are responsible for the formation of crystallin adducts and cross-links in vivo [2]. In this process, dehydroascorbic acid (DHA) and its degradation products 2,3-diketogulonic acid (DKG), xylosone, and erythrulose can act as precursors of the lysine-arginine crosslink pentosidine, the lysine-lysine cross-links vesperlysine A and K2P, the arginine hydroimidazolone of glyoxal (G-H1) and methylglyoxal (MG-H1), and the lysine adducts carboxymethyl- and carboxyethyl-lysine (CML, CEL). Examples are shown in Figure 1. All these modifications are present in the aging human lens [3] and could be duplicated in the transgenic mice that expresses the human vitamin C transporter 2 (hSVCT2) in their lens [2] and in vitro [4,5]. In these mice the ascorbate levels are as high as those present in the human lens, i.e., 1–3 mM.

While there is ample in vitro evidence for the fact that crystallin ascorbylation and glycation can be deleterious on the lens crystallin structure and function [6-8], the critical questions are 1) whether these can be pharmacologically prevented, and 2) whether prevention of the latter delays the progression of age-related nuclear sclerosis. Toward the first question, we have previously tested the ability of several orally administered candidate pharmacological compounds to block ascorbylation in the hSVCT2 mouse lens, i.e., the guanidino compounds NC-1 (L-arginine) and NC-2 (γ-guanidinobutyric acid), aminoguanidine, the sulfhydryl compound penicillamine, and the nucleophilic scavenger pyridoxamine. The compound with the most consistent activity was NC-1, i.e., L-arginine fed at the concentration of 0.1% (wt/wt) in food [9]. All tested modifications were decreased by 25%–65%.

As a preamble to a clinical trial, we now have investigated whether topical application for seven months of L-arginine, γ-guanidinobutyric acid, *N*-acetylcysteine, and penicillamine (a copper chelator) to one eye of hSVCT2 mice can block crystallin ascorbylation compared to the control eye. This demonstration is essential since the most desirable clinical outcome would be to demonstrate that age-related modifications and nuclear sclerosis can be delayed using topical instead of systemic application. We have also tested whether L-arginine can also be taken up into the rabbit lens in vitro as well as when applied topically to the eye.

## Methods

### Experimental animals

All animal experiments were conducted in accordance with procedures approved by the Case Western Reserve University, Cleveland, OH Animal Care Committee and conformed to the ARVO Statement for use of Animals in Ophthalmic and Vision Research. Animals were housed under diurnal lighting condition and allowed free access to food and water. hSVCT2 transgenic mice were maintained as described previously [2]. The genetic background of these transgenic mice is C57BL/6 after multiple rounds of breeding with this strain.

### Eye drop formulation and topical application to hSVCT2 mouse eye

L-Arginine (A5131), DL-penicillamine (P5125), γ-guanidinobutyric acid (G6503), and *N*-acetyl-L-cysteine (N7250) were all purchased from Sigma Company (St. Louis, MO). Solutions containing 0.1% of inhibitor were produced in phosphate buffer saline (PBS) whereby the pH was adjusted to 7.4 as needed and sterilized over 0.2 µm filters. The solutions were aliquoted into 1 ml fractions and stored in a −80 °C freezer. hSVCT2 transgenic mice (n=10) received twice per day, 5 days per week starting at 2 months and continued till 9 months of age, one eye drop of inhibitor in the right eye and vehicle control in the left eye. Mice were sacrificed and eyes were removed and decapsulated to release the lenses. These were processed for advanced glycation end product (AGE) determination as described below.

### Rabbit lens topical inhibitors kinetic study

Two rabbits in each group received one drop of a solution of 0.5% inhibitor dissolved in Systane Lubricant Eye Drops (Alcon, Fort Worth, TX) on the right eye and 2% inhibitor similarly prepared on the left eye. The rabbits were sacrificed at various time points and the lenses were quickly dissected and washed three times with ice-cold PBS. The lenses were then homogenized in 1.0 ml ice-cold PBS, centrifuged at 20,000× g for 25 min and the supernatant was used to determine the content of inhibitors using liquid chromatography/mass spectrometry (LC/MS).

### In vitro incubation of rabbit lenses with L-arginine and N-acetylcysteine

Freshly excised rabbit lenses were preincubated for 8 h in Dulbecco’s modified Eagle’s medium (Medium 199; Sigma Company) to verify viability, and further incubated with or without added 10 mM L-arginine or *N*-acetylcysteine in the presence of 25 mM glucose, or 5 mM ascorbate, or 100 µM dehydroascorbate. After 24 h, lenses were processed to determine the uptake of L-arginine or N-acetylcysteine (NAC).

### Processing of mice lenses for the determination of protein-bound AGEs

Lenses were homogenized in ice-cold 10% trichloracetic acid (TCA), and placed on ice for 15 min. The TCA protein precipitate was washed twice with 500 μl of ethyl ether and further delipidated with 500 μl of chloroform/methanol (2:1) at 4 °C overnight, then soaked in water and lyophilized. The lyophilized sample representing on average 1 mg protein was reconstituted with 500 μl of 5.0 mM argon-exchanged, Chelex-treated phosphate buffer (pH 7.0) by sonication. The suspension was solubilized with 35 µl of 10 mg/ml protease K at 37 °C for 24 h for determination of protein-bound fluorescence. The sample was then divided into two equal fractions, one of which was subjected to further enzymatic digestion, and the other subjected to hydrolysis with 6 N HCl following our previous described method [2] to release free AGEs.

### Fluorescence spectroscopy and advanced glycation endproducts determination

The fluorescence at λex/em 370/440 nm and 335/385 nm of the enzymatic lens protein digest was measured with a spectrofluorometer (821-F; Jasco, Easton, MD). The data was expressed as fluorescence units per unit protein measured as leucine equivalent. Carboxymethyl-lysine (CML) and carboxyethyl-lysine (CEL) were determined by the gas chromatography/mass spectrometry (GC/MS) method and the hydroimidazolones G-H1 and MG-H1, and glucosepane were determined by the LC/MS method as described in our previous study [10].

### Determination of L-arginine and *N*-acetylcysteine

Uptake of L-arginine and *N*-acetylcysteine was determined in the protein-free rabbit lens extract using the same method as for the rabbit lens topical inhibitor study (see above). Arginine was assayed by isotope dilution technique using ^15^N-arginine as an internal standard. The *m/z* transition 174.9>70.1 and 176.9>70.2 were used for arginine and ^15^N-arginine, respectively. Cone voltage (Cv) and collision energies (Ce) were 60 V/22 eV and 60 V/20 eV, respectively. *N*-acetylcysteine was quantified based on the peak area for the transition *m/z* 163.66x>121.85 (loss of acetyl; Cv:48 V, CE: 9 eV, respectively) in the same chromatographic analysis run as for L-arginine.

### Statistical analysis

All values were expressed as means±SD. Statistical significance of the differences in mean values was assessed by repeated-measures of ANOVA or Student’s *t*-test. P values of <0.05 were considered statistically significant.

## Results

**Figure 2:**
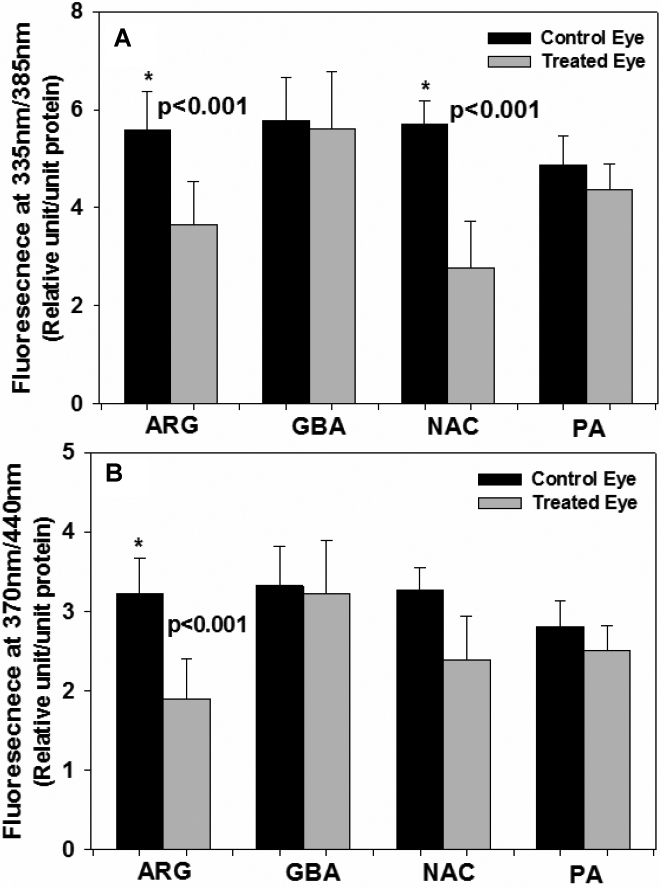
Levels of protein-bound fluorescence in transgenic mouse lens protein with and without inhibitor treatment. **A**: Fluorescence at λ_ex/em_ 335/385 nm and **B**: Fluorescence at λ_ex/em_ 370/440 nm. One-way ANOVA was used followed by post-hoc analysis for all comparisons (n=10 per group). L-Arginine (ARG) significantly reduced fluorescence at 335/385 nm (p<0.001) and 370/440 nm (p<0.001). *N*-acetylcysteine (NAC) significantly reduced the fluorescence at 335/385 nm (p<0.001). GBA=guanidinobutyric acid, PA=penicillamine.

**Figure 3:**
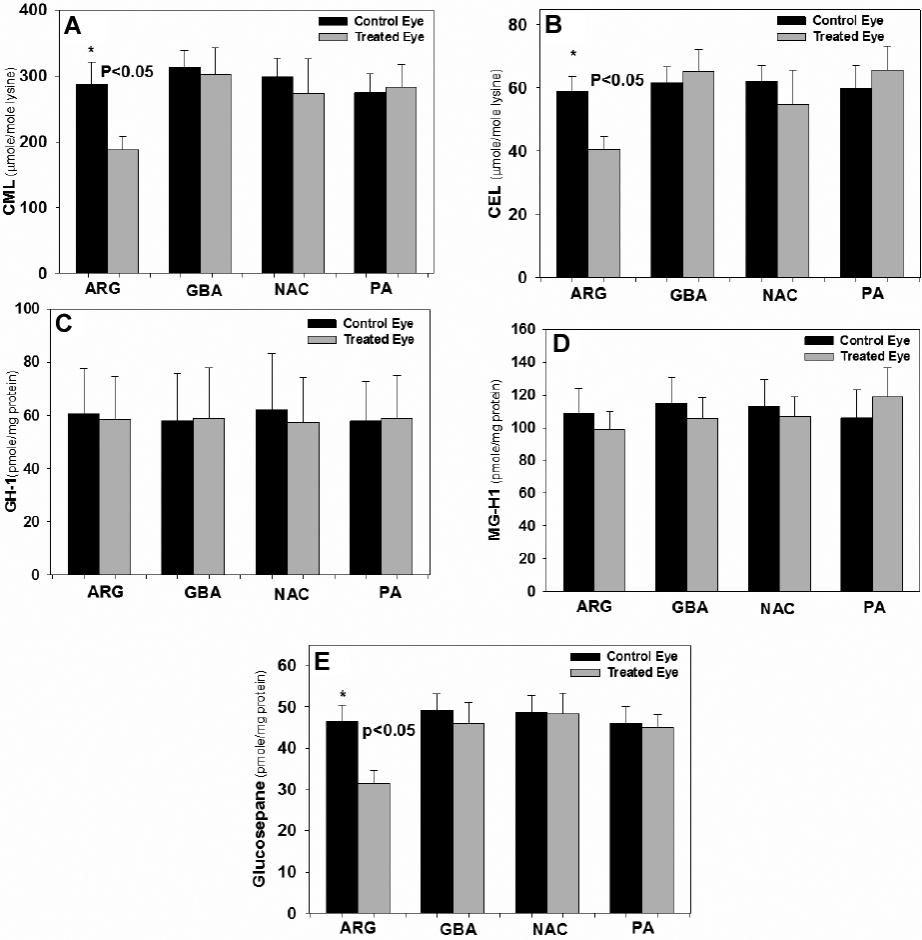
Additional AGE levels in mouse lens with or without inhibitor eye drop treatment. **A**: Mouse lens protein CML levels were significantly reduced by L-arginine (p<0.05). **B**: Mouse lens protein CEL levels were significantly reduced by L-arginine (p<0.05). **C**: Mouse lens protein GH1 were not affected by inhibitors (p=N.S.) versus vehicle control. **D**: Mouse lens protein MG-H1 levels were not affected by inhibitors (p=N.S) versus vehicle control. **E**: Mouse lens protein glucosepane levels were significantly reduced by L-arginine (p<0.05). One-way ANOVA was used followed by post-hoc analysis for all comparisons (n=10 per group). For abbreviations, see Figure 1.

One drop of potential inhibitors, each 0.1% dissolved in phosphate buffer saline of L-arginine, guanidinobutyric acid, DL-penicillamine, or *N*-acetylcysteine was applied five days a week to the right eye of hSVCT2 mice. The contralateral eye received vehicle only, i.e., PBS. Total lens crystallins were isolated, enzymatically digested, and processed for the measurement of protein-bound fluorescence at 335/385 nm and 370/440 nm and advanced glycation/ascorbylation products as previously described [10]. After seven months, L-arginine suppressed pentosidine-like fluorescence at 335/385 nm and 370/440 nm fluorescence by 40% (p<0.001; Figure 2). Interestingly, the latter was also 50% suppressed by *N*-acetylcysteine (p<0.05; Figure 2). NAC suppressed 335/385 fluorescence, though not significantly. L-Arginine also suppressed CML, CEL, and glucosepane cross-links by 35% (p<0.05), 30% (p<0.05) and 37% (p<0.05), respectively (Figure 3A,B,E). Surprisingly it did not suppress the methylglyoxal hydroimidazolone MG-H1 and the glyoxal hydroimidazolone G-H1 (Figure 2C,D). Except for the positive effect of NAC on 335/385 nm fluorescence (Figure 2A), neither the latter nor penicillamine (PA) or guanidinobutyric acid (NAC-2) had any effect on any of the advanced glycation endproducts (Figure 2 and Figure 3).

**Figure 4:**
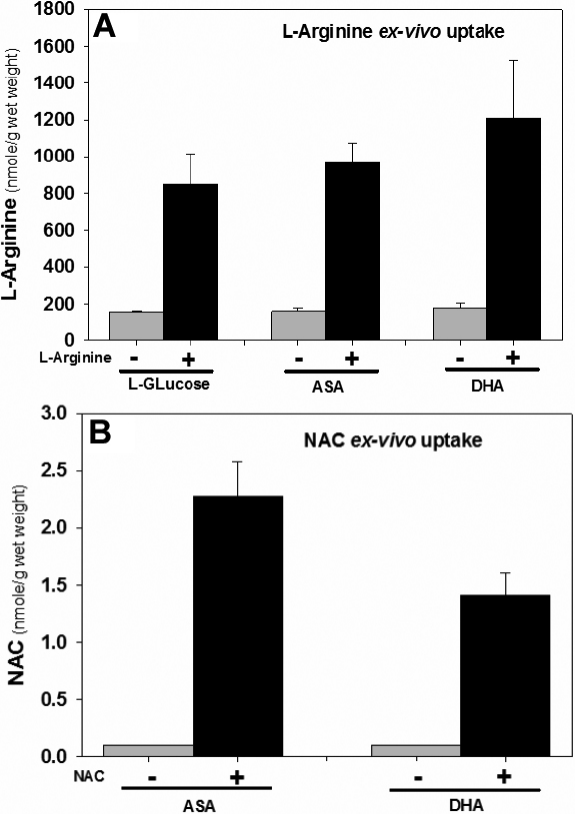
Comparative uptake of L-arginine and *N*-acetyl-L-cysteine in rabbit lenses (n=2) incubated with 5 mM L-arginine and 5 mM NAC with and without presence of 25 mM D-glucose or 5 mM ascorbic acid and 0.1 mM dehydroascorbic acid for 4 h. The lenses were washed with cold PBS and homogenized in water for L-arginine and NAC determination in supernatant by LC/MS.

The above findings suggest that mice have the ability to take up L-arginine and *N*-acetylcysteine trans-corneally. To find out if this was potentially applicable to other species, we determined the uptake in vitro and in vivo of L-arginine in rabbit lenses upon transcorneal application. Lenses were incubated with 5 mM concentration of L-arginine in ascorbic acid, dehydroascorbic acid (DHA) or D-glucose in Dulbecco’s modified Eagle’s medium 199 for 24 h under different conditions to simulate either the ascorbate or glucose concentration of the medium. The chosen concentration (i.e., 5 mM L-arginine) was five times lower than that applied to the hSVCT2 mouse eye. i.e., 0.5% or 28 mM. The results were compared with 5 mM *N*-acetylcysteine (NAC) incubated under similar conditions. As shown in Figure 4, lenticular arginine levels in the absence of added L-arginine varied from 150 to 210 nmol/g wet weight (mean±SD: 166.5±17.5 nmol/g wet weight, n=6) and jumped to values ranging from 780 to 1,432 nmol/g (mean±SD 1,008.7±233.5 nmol/g) when lenses were incubated with 5 mM arginine. This increase was highly significant (p<0.0001). For comparison, NAC levels were 1.60±0.85 nmol/g wet weight (n=4) in the presence of 5 mM added NAC, while no NAC was detected in lenses incubated without added NAC.

**Figure 5:**
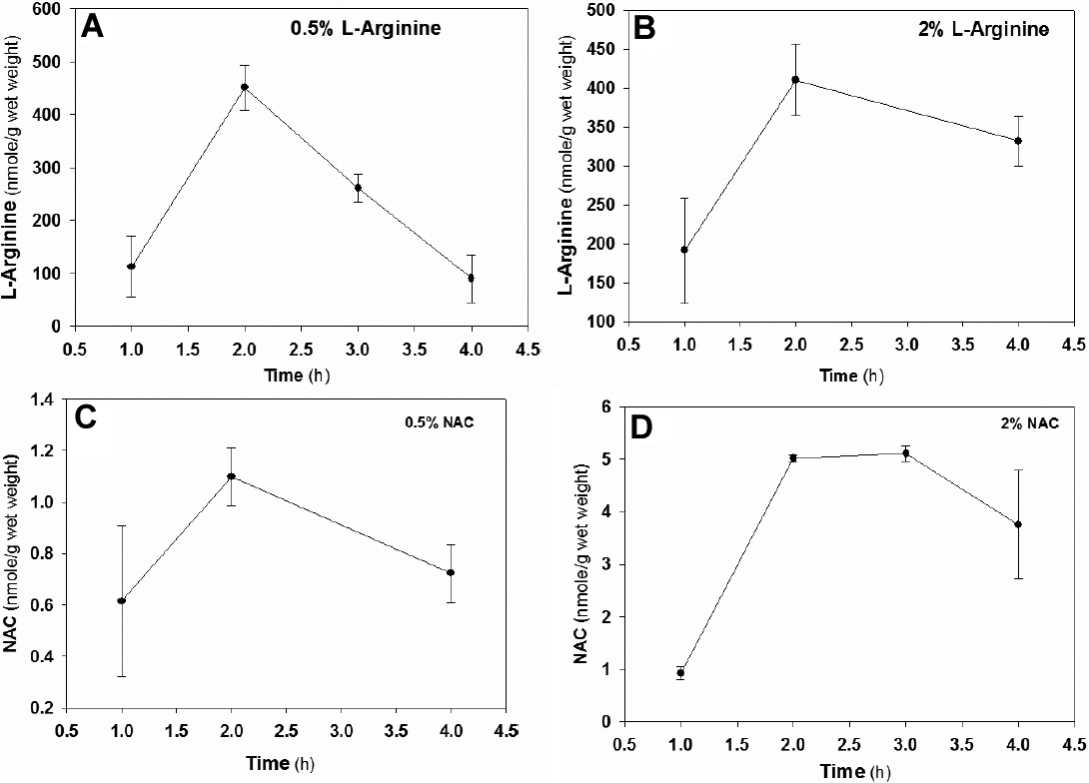
Uptake kinetics of L-Arginine and NAC in rabbit lens. Two rabbits in each group were topically applied with 0.5% inhibitor on right eye and 2% inhibitor on the left eye. The inhibitors were quantified by LC-MS in total lens extract. **A**: 0.5% L-arginine eye drop; **B**: 2% L-arginine eye drop; **C**: 0.5% NAC eye drop; **D**: 2% NAC eye drop.

Finally, lenticular uptake of L-arginine upon in vivo transcorneal application to the rabbit eye showed a rapid transcorneal uptake which reached similar lenticular plateau levels varying from 400 to 500 nmol/g after 120 min, regardless of whether 0.5 or 2.0% eye drops were applied (Figure 5). However the latter concentration remained more elevated at 4 h in presence of 2.0% compared to 0.5%. Similarly, NAC levels reached a plateau at 2 h, but levels were three to four times higher and persisted longer with 2.0% instead of 0.5% NAC.

## Discussion

The above results confirm the ability of L-arginine (previously referred to as “NC-1” [10]) to suppress the formation of advanced ascorbylation end products in the hSVCT2 mouse model of human lens brunescence. Quite remarkably, except for the fact that NAC lowered the fluorescence at 335/385 nm, L-arginine was the only compound able to significantly delay the accumulation of multiple AGEs.

We tentatively attribute the ability of arginine to suppress AGE formation to its ability to scavenge oxoaldehydes such as glyoxal and methylglyoxal, as well as DHA and its degradation products xylosone, erythrulose, and deoxythreosone. While γ-guanidinobutyric acid had expectedly similar effects when fed orally to hSVCT2 mice, no such effects were noted in the present experiments. We attribute this discrepancy to the fact that specific transporters for L-arginine must be present in the cornea and lens, which preferentially favor its uptake over γ-guanidinobutyric acid into the lens. When orally fed at 0.1% in the diet, presumably sufficient concentrations of the latter are achieved to allow scavenging of oxoaldehydes. Indeed Jain-Vakkalagadda et al. [10] found that transport of L-arginine across rabbit cornea was saturable (K_m_=306±72 μM and V_max_=0.12±0.01 nmol min^−1^cm^−2^) and was Na^+^, Cl^-^, and energy dependent, and inhibited by neutral and cationic amino acids. The specific B(0,+) arginine transporter was identified in both rabbit and human corneas.

Concerning the uptake of arginine into the lens, numerous studies on the uptake of various amino acids into the animal and human lens have been done in the past [11-14]. One study suggests a ~1:1 ratio of lens: aqueous arginine levels [11], which would exclude the presence of high affinity uptake into the lens. However the aqueous: plasma ratio was ~3:1 implying active uptake from the plasma into the aqueous. Systematic studies on arginine transport are lacking and molecular studies are needed to clarify this field.

L-Arginine is an attractive drug for the potential delay of senile cataracts for multiple reasons. First, recent studies from our laboratory have unequivocally demonstrated that arginine is the single major damaged crystallin residue by advanced glycation during aging [3]. In addition, various studies by others have demonstrated the ability of arginine to block in vitro and in vivo advanced glycation [15-22]. Second, recent studies also suggest that arginine added to proteins in solution has stabilizing effects by the prevention of aggregation and improving solubilization [23,24]. Thus, topically applied arginine might improve the progression of cataractogenesis by acting as an AGE inhibitor and a protein stabilizing agent. Finally and importantly, L-arginine has low toxicity since it is a natural constituent.

Another important aspect of the above study is the demonstration that *N*-acetylcysteine was able to block protein bound formation of AGE fluorescence. The latter might be a consequence of UV or other oxidation mediated damage to tryptophan residues, rather than a glycation-related process [25]. NAC is widely used for suppression of oxidant stress in cell culture experiments, and is an approved clinical drug of low toxicity for the treatment of acetaminophen poisoning and various other conditions [26].

In summary, the above study confirms the in vivo potential of L-arginine as a blocking agent of carbonyl stress in the lens. In addition, our finding that topical application of NAC was potent at decreasing protein-bound fluorescence at 335/385 nm, and to some extent at 370/440 nm, suggests it might be useful as an adjuvant to L-arginine for combating combined carbonyl and oxidant stress in the pathogenesis of age-related nuclear cataracts.

## References

[1] Sharma KK, Santhoshkumar P (2009). Lens aging: Effects of crystallins.. Biochim Biophys Acta.

[2] Fan X, Reneker LW, Obrenovich ME, Strauch C, Cheng R, Jarvis SM, Ortwerth BJ, Monnier VM (2006). Vitamin C mediates chemical aging of lens crystallins by the Maillard reaction in a humanized mouse model.. Proc Natl Acad Sci USA.

[3] Fan X, Sell DR, Zhang J, Nemet I, Theves M, Lu J, Strauch C, Halushka MK, Monnier VM (2010). Anaerobic vs aerobic pathways of carbonyl and oxidant stress in human lens and skin during aging and in diabetes: A comparative analysis.. Free Radic Biol Med.

[4] Cheng R, Feng Q, Argirov OK, Ortwerth BJ (2004). Structure elucidation of a novel yellow chromophore from human lens protein.. J Biol Chem.

[5] Cheng R, Lin B, Lee KW, Ortwerth BJ (2001). Similarity of the yellow chromophores isolated from human cataracts with those from ascorbic acid-modified calf lens proteins: evidence for ascorbic acid glycation during cataract formation.. Biochim Biophys Acta.

[6] Linetsky M, Shipova E, Cheng R, Ortwerth BJ (2008). Glycation by ascorbic acid oxidation products leads to the aggregation of lens proteins.. Biochim Biophys Acta.

[7] Nagaraj RH, Oya-Ito T, Padayatti PS, Kumar R, Mehta S, West K, Levison B, Sun J, Crabb JW, Padival AK (2003). Enhancement of chaperone function of alpha-crystallin by methylglyoxal modification.. Biochemistry.

[8] Abraham EC, Huaqian J, Aziz A, Kumarasamy A, Datta P (2008). Role of the specifically targeted lysine residues in the glycation dependent loss of chaperone activity of alpha A- and alpha B-crystallins.. Mol Cell Biochem.

[9] Fan X, Monnier VM (2008). Inhibition of crystallin ascorbylation by nucleophilic compounds in the hSVCT2 mouse model of lenticular aging.. Invest Ophthalmol Vis Sci.

[10] Jain-Vakkalagadda B, Pal D, Gunda S, Nashed Y, Ganapathy V, Mitra AK (2004). Identification of a Na+-dependent cationic and neutral amino acid transporter, B(0,+), in human and rabbit cornea.. Mol Pharm.

[11] Reddy DV (1965). Amino Acid Transport in the Lens in Relation to Sugar Cataracts.. Invest Ophthalmol.

[12] Reddy DV, Kinsey VE (1963). Transport of amino acids into intraocular fluids and lens in diabetic rabbits.. Invest Ophthalmol.

[13] Reddy DV, Kinsey VE, Nathorst-Windahl G (1966). Comparison of amino acid transport in ocular structures of rabbits made diabetic by alloxan and pancreatectomy.. Invest Ophthalmol.

[14] Reddy DVN, Kinsey VE (1963). Transport of amino acids into intraocular fluids and lens in diabetic rabbits.. Invest Ophthalmol.

[15] Méndez JD, Balderas FL (2006). Inhibition by L-arginine and spermidine of hemoglobin glycation and lipid peroxidation in rats with induced diabetes.. Biomed Pharmacother.

[16] Méndez JD, Leal LI (2004). Inhibition of in vitro pyrraline formation by L-arginine and polyamines.. Biomed Pharmacother.

[17] Servetnick DA, Bryant D, Wells-Knecht KJ, Wiesenfeld PL (1996). L-Arginine inhibits in vitro nonenzymatic glycation and advanced glycosylated end product formation of human serum albumin.. Amino Acids.

[18] Weninger M, Xi Z, Lubec B, Szalay S, Hoger H, Lubec G (1992). L-Arginine reduces glomerular basement membrane collagen N-Carboxymethyllysine in the diabetic db-db mouse.. Nephron.

[19] Selwood T, Thornalley PJ (1993). Binding of methylglyoxal to albumin and formation of fluorescent adducts. Inhibition by arginine, N-acetylarginine and aminoguanidine.. Biochem Soc Trans.

[20] Radner W, Hoger H, Lubec B, Salzer H, Lubec G (1994). L-arginine reduces kidney collagen accumulation and N-epsilon-(carboxymethyl)lysine in the aging NMRI-mouse.. J Gerontol.

[21] Pischetsrieder M (1996). Reaction of L-ascorbic acid with L-arginine derivatives.. J Agric Food Chem.

[22] Lubec B, Aufricht C, Amann G, Kitzmuller E, Hoger H (1997). Arginine reduces kidney collagen accumulation, cross-linking, lipid peroxidation, glycoxidation, kidney weight and albuminuria in the diabetic kk mouse.. Nephron.

[23] Arakawa J, Uegaki M, Ishimizu T. (2011). Effects of l-arginine on solubilization and purification of plant membrane proteins.. Protein Expr Purif.

[24] Arakawa T, Kita Y, Ejima D, Tsumoto K, Fukada H (2006). Aggregation suppression of proteins by arginine during thermal unfolding.. Protein Pept Lett.

[25] Baynes JW, Thorpe SR (1999). Role of oxidative stress in diabetic complications: a new perspective on an old paradigm.. Diabetes.

[26] Dodd S, Dean O, Copolov DL, Malhi GS, Berk M (2008). N-acetylcysteine for antioxidant therapy: pharmacology and clinical utility.. Expert Opin Biol Ther.

